# Functional and mechanistic characterization of the phosphatidyl-*myo*-inositol mannosyltransferase PimA from *Mycobacterium tuberculosis*: implications for anti-tuberculosis drug development

**DOI:** 10.3389/fcimb.2026.1888373

**Published:** 2026-08-19

**Authors:** Dafeng Liu, Huashui Deng, Xinbao Liu, Wenshuang Yao, Na Li

**Affiliations:** 1 College of Biological Sciences and Technology, Yili Normal University, Yining, Xinjiang, China; 2 School of Life Sciences, Xiamen University, Xiamen, Fujian, China

**Keywords:** enzyme activity assays, gene expression profiles, *Mycobacterium tuberculosis*, phosphatidyl-*myo*-inositol mannosyltransferase PimA, the molecular docking

## Abstract

Human-adapted *Mycobacterium tuberculosis* (*Mtb*) also infects animals and transmits zoonotically, threatening public health. The cell wall glycolipids lipoarabinomannan and lipomannan are essential virulence factors of *Mtb*. Phosphatidyl-*myo*-inositol mannosyltransferase PimA catalyzes a key step in their biosynthesis and represents a potential drug target. However, the functional mechanism of *Mtb* PimA remains incompletely understood. Here, the hydrodynamic diameter of monomeric PimA was measured as 5.1 ± 0.2 nm. The structural model of PimA was predicted using AlphaFold2. Molecular docking was conducted to predict potential binding sites, and site-directed mutagenesis was then performed. Mutations D253A, E274A or E282A dramatically reduced the activity, whereas R196A, K202A or K256A abolished activity entirely. Guanosine diphosphate (GDP) and benzimidazole inhibited PimA by over 50% at 40 µM and 10 µM, respectively. PimA transcription increased linearly over time in macrophages, and conditional PimA-silenced mutant rendered *Mtb* rapidly susceptible to macrophage killing. These results provide a structural and functional framework for the rational design of novel anti-tubercular agents targeting PimA.

## 1 Introduction

*Mycobacterium tuberculosis* (*Mtb*), the primary etiological agent of human tuberculosis (TB), has traditionally been considered a human-adapted pathogen (Trajman et al., 2025; Warner et al., 2025; Dartois et al., 2025). However, accumulating evidence supports its role in zoonotic transmission, challenging the classical host restriction paradigm and raising public health concerns globally (Goig et al., 2025; Warner et al., 2025; Liu et al., 2025a). The burden of TB is compounded by co-morbidities such as HIV and SARS-CoV-2 infections (Russell et al., 2025; Schmidt et al., 2025). The emergence and global spread of drug-resistant *Mtb* strains, including resistance to isoniazid, pyrazinamide, and rifampicin (Dominguez et al., 2023; Naidoo and Dookie, 2022; Dean et al., 2022), further undermine treatment efficacy and drive high mortality rates (Tiberi et al., 2018; Micoli et al., 2021; Lange et al., 2019; Jenkins et al., 2014; Getahun et al., 2015; Dheda et al., 2017; Furin et al., 2019; Reid et al., 2019; Liu et al., 2025b; Liu and Li, 2026b). These challenges underscore the urgent need to revisit fundamental drug discovery approaches, emphasizing the identification of new therapeutic targets and the development of novel anti-tubercular agents.

The extraordinary resilience and intrinsic drug resistance of *Mtb* are largely due to its distinctive, complex cell envelope (Dartois et al., 2025; Goig et al., 2025). Beyond the plasma membrane and peptidoglycan layer lies a robust outer barrier enriched in atypical lipids and glycolipids (Guerin et al., 2009b; Boldrin et al., 2014; Rodrigo-Unzueta et al., 2016). Among these, lipoarabinomannan (LAM) and its precursor lipomannan (LM) are critical virulence factors that enable *Mtb* to survive within the hostile environment of host macrophages (Guerin et al., 2009b; Boldrin et al., 2014; Rodrigo-Unzueta et al., 2016). These phosphatidylinositol-anchored glycolipids modulate host immunity by inhibiting phagosome maturation, scavenging reactive oxygen species, and altering cytokine responses (Guerin et al., 2009b; Boldrin et al., 2014; Rodrigo-Unzueta et al., 2016). Accordingly, the biosynthetic pathways of LAM and LM provide a valuable source of drug targets absent in humans.

**Figure 1:**
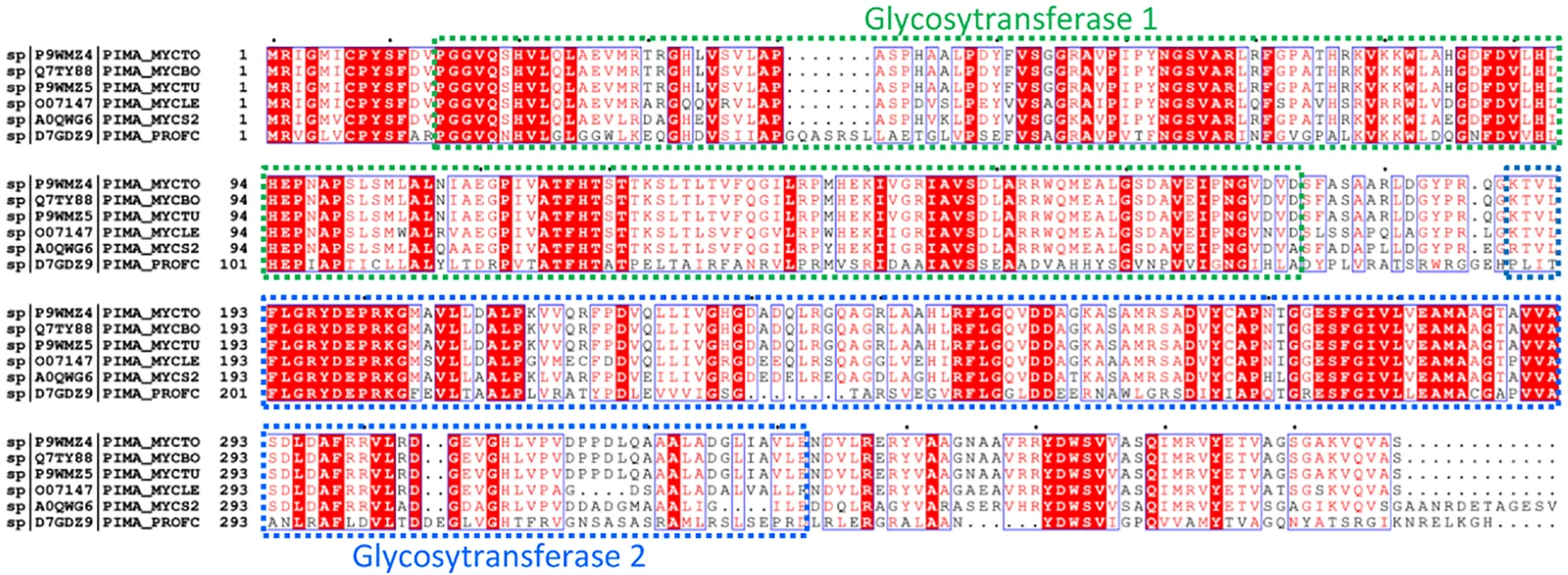
Sequence alignment of phosphatidyl-myo-inositol mannosyltransferase family. The ClustalW default color scheme was applied, with conserved amino acids indicated by more intense coloring than non-conserved residues. The alignment incorporates the following reference sequences: P9WMZ4, *Mycobacterium tuberculosis* (strain CDC 1551/Oshkosh); Q7TY88, *Mycobacterium bovis* (strain ATCC BAA-935/AF2122/97); P9WMZ5, *Mycobacterium tuberculosis* (strain ATCC 25618/H37Rv); O07147, *Mycobacterium leprae* (strain TN); A0QWG6, *Mycolicibacterium smegmatis* (strain ATCC 700084/mc(2)155); D7GDZ9, *Propionibacterium freudenreichii subsp. shermanii* (strain ATCC 9614/DSM 4902/CIP 103027/NCIMB 8099/CIRM-BIA1). Glycosyltransferase 1 and glycosyltransferase 2 were colored green and blue, respectively. In a “*” within a sequence alignment indicates a gap. This gap represents an insertion or deletion (indel) introduced by the alignment algorithm to maximize overall residue matching across all sequences. At that specific position, the given sequence lacks a corresponding amino acid compared to the other sequences.

The first committed step in the biosynthesis of LM and LAM is catalyzed by phosphatidyl-*myo*-inositol mannosyltransferase PimA (Guerin et al., 2009b; Boldrin et al., 2014; Rodrigo-Unzueta et al., 2016). PimA transfers a mannose residue from GDP-Man to the 2-position of the myo-inositol ring of phosphatidyl-myo-inositol (PI), producing phosphatidyl-myo-inositol monomannoside (PIM_1_) (Tatituri et al., 2007; Guerin et al., 2009a; Batt et al., 2010). This initial glycosylation serves as the foundational building block for higher-order PIMs, LM, and LAM, and functions as a critical metabolic checkpoint. PimA is essential for *Mtb* growth *in vitro*, underscoring its non-redundant physiological role. Structurally, PimA belongs to the GT-A fold family of glycosyltransferases, requires a divalent metal ion for activity, and features a conserved DXD motif central to its catalytic mechanism (**Figure 1**). However, the functional and mechanistic details of *Mtb* PimA remain poorly understood.

Herein, the monomeric form of PimA exhibited a hydrodynamic diameter of 5.1 ± 0.2 nm. Molecular docking was performed based on the AlphaFold2-predicted model to predict potential binding sites, and then site-directed mutagenesis was conducted. While mutations D253A, E274A, or E282A substantially decreased enzymatic function, substitutions R196A, K202A, or K256A resulted in a complete loss of activity. PimA was inhibited by more than 50% in the presence of 40 µM guanosine diphosphate (GDP) and 10 µM benzimidazole, respectively. Transcript levels of PimA increased linearly within macrophages over time. Furthermore, conditional PimA-silenced mutant made *Mtb* highly vulnerable to macrophage killing, underscoring the enzyme’s essential role for intracellular survival. These results clarify the mechanism of PimA and create a platform for the structure-guided development of new anti-tuberculosis compounds that target this critical enzyme in the *Mtb* cell wall biosynthesis pathway.

## 2 Materials and methods

### 2.1 PimA constructs, expression and purification

Constructs, expression, and purification of PimA (UniProt P9WMZ5) were performed based on previously reported methods with modifications (Gu et al., 2005; Boldrin et al., 2014, 2021) (**Supplementary Figures 1, 2**). The PimA-pET28a plasmid was used to transform *E. coli* BL21 (DE3) cells. Transformed cells were initially grown at 37 °C in LB medium containing 50 µg/mL kanamycin for 12 h until the optical density at 600 nm (OD _600 nm_) reached 2. A 10 mL culture was then inoculated into 1 L LB medium with kanamycin (50 µg/mL) and incubated at 37 °C for ~2 h until OD _600 nm_ reached 0.6-0.8. The culture was cooled to 20 °C over 30 min, and protein expression was induced with 0.5 mM IPTG (Isopropyl-beta-D-thiogalactopyranoside) for 18 h at 20 °C. Cells from 1 L culture were harvested by centrifugation at 5000 rpm for 15 min at 4 °C and resuspended in 40 mL buffer A (50 mM NaH_2_PO_4_, pH 7.4; 150 mM NaCl; 10 mM imidazole). Cells were lysed on ice by sonication at 120 W for four cycles of 60 s pulses with 2 s rests between bursts. The lysate was clarified by centrifugation at 18,000 rpm for 30 min at 4 °C. A Ni-NTA affinity column (4 mL resin) was pre-equilibrated with 10 column volumes (CV) of buffer A. The supernatant was loaded by gravity, and the column was washed with 25 CV of buffer B (50 mM NaH_2_PO_4_, pH 7.4; 150 mM NaCl; 40 mM imidazole; 5% glycerol). PimA was eluted with 1.5 CV of buffer C (50 mM NaH_2_PO_4_, pH 7.4; 150 mM NaCl; 300 mM imidazole).

### 2.2 Dynamic light scattering experiments

The oligomeric state of PimA was evaluated by dynamic light scattering (DLS) using a Dynapro DLS instrument (Malvern Zetasizer, Malvern, UK). PimA was concentrated to 2.4 mg/mL and centrifuged at 18,000 rpm for 5 min at 4 °C. Samples were loaded into a 1 cm path-length cuvette, and data were collected over 30 runs with a 120 s equilibration per run. DLS measurements were analyzed using Zetasizer software (v6.20), which generated regularized histograms to determine the hydrodynamic diameter throughout the analysis.

### 2.3 Prediction and evaluation of the structural model of PimA

The PimA structural model was predicted using AlphaFold2 (Wayment-Steele et al., 2023; Jumper et al., 2021) based on its protein sequence (UniProt P9WMZ5; **Supplementary Figures 1-3**). Molecular graphics were visualized with PyMOL v2.3.4 (https://www.pymol.org/2/). Structural validation was performed via geometric analysis: a Ramachandran plot generated using PDBsum (Laskowski, 2022; de Beer et al., 2014; Laskowski, 2004, 2009; Laskowski et al., 2017) assessed backbone dihedral angles (φ/ψ) to evaluate stereochemical quality, identifying favorable conformations and flagging energetically unfavorable residues, thereby confirming the reliability of the predicted model. Additional validation was conducted using ProSA, a widely adopted tool for assessing protein structures from X-ray and NMR data (Wiederstein and Sippl, 2007).

Multiple sequence alignments were performed using ClustalW with default parameters (Gouet, 2003; Larkin et al., 2007), and the results were visualized with ESPript 3.0 (Robert and Gouet, 2014).

### 2.4 Molecular docking

The binding sites were predicted via molecular docking of GDP (guanosine diphosphate) and GDD (GDP-α-D-mannose) using AutoDockTools 1.5.7 and AutoDock 4.2.6 (Huey et al., 2007; Morris et al., 2009; Trott and Olson, 2009; Forli et al., 2016). The Genetic Algorithm parameters were set to 1000 runs, a population size of 200, 30,000 generations, and 3,000,000 evaluations. Docking results were analyzed and visualized with PyMOL v2.3.4 (https://www.pymol.org/2/). The calculated binding energy of -6.12 kcal/mol suggested a ligand-protein affinity, but this result should be interpreted as a predictive indication rather than a confirmation of the docking reliability. Binding energy, expressed in kcal/mol, reflects ligand-protein interaction strength in computational models, with more negative values generally corresponding to stronger predicted binding and greater complex stability. This parameter can therefore serve as a predictive metric for evaluating potential functional modulation and for guiding the selection of bioactive compounds for further study.

### 2.5 Enzymatic activity assay

Enzymatic activity of PimA and its mutants (**Supplementary Table 1**) was measured using a modified spectrophotometric assay based on previously described methods (Gu et al., 2005; Raman et al., 2008; Boldrin et al., 2014, 2021). Reactions were performed at 37 °C and monitored by NADH absorption at 340 nm using a microplate reader (Synergy 4, BioTek Instruments). Each 100 µL reaction contained PimA reaction buffer (50 mM HEPES, pH 7.4; 3 mM MgCl_2_), 0.5 mM phospho(enol)pyruvate (PEP), 10 U lactic dehydrogenase, 5 U pyruvate kinase, 0.25 mM NADH, 67 µM GDP-Man, 200 µM bovine PI, and PimA at 0.2 mg/mL. All components except the substrate and PimA were premixed, then the substrate was added and incubated for 10 min at 37 °C to stabilize the background before initiating the reaction with PimA. One unit of activity was defined as 1 µmol NAD^+^ formed per minute per milligram of enzyme. Reactions were monitored for 10 min, with data corrected for non-enzymatic background. Michaelis constant (*K_m_*) and catalytic constant (*K_cat_*) were determined using Hanes-Woolf plots. Inhibitory effects of GDP and benzimidazole at varying concentrations were evaluated by including them in the reaction mixtures.

### 2.6 Assay of enzymatic activity in PimA site-directed mutants

Primers for PimA site-directed mutagenesis were designed for PCR and synthesized by Shanghai Sangon Biotechnology (China) (**Supplementary Table 1**; **Supplementary Figures 1, 2**). The pET28a plasmid containing the PimA gene served as the template, and mutagenesis was performed using Q5 polymerase (**Supplementary Table 1**; **Supplementary Figures 1, 2**). Successful mutations were confirmed by nucleotide sequencing (Shanghai Sangon Biotechnology, China). The mutated PimA genes were cloned back into the pET28a vector, and expression and purification followed the same protocols as for wild-type (WT) PimA (**Supplementary Figure 4**). Enzymatic assays of the mutant proteins were conducted under identical conditions to those used for WT PimA.

In the mutation experiment, all target residues were replaced by alanine. From a structural standpoint, alanine is widely regarded as the substitution of choice because it eliminates side-chain chemistry while typically causing minimal backbone perturbation (Weiss et al., 2000; Cunningham and Wells, 1989). The mutation truncates the native residue at the β-carbon, removing all atoms beyond Cβ and thereby abolishing hydrogen bonds, salt bridges, and hydrophobic packing interactions. Importantly, retaining the β-carbon helps preserve the local backbone dihedral angles (φ/ψ) and restricts conformational entropy, which is expected to maintain the global fold without backbone collapse, a significant advantage over glycine, which lacks a β-carbon and can introduce unwanted flexibility. The high α-helical propensity of alanine also tends to prevent secondary-structure destabilization, making helix breaks or β-sheet shifts less likely. Consequently, the post-mutation structure is likely to remain largely isosteric with the wild-type backbone. Based on this property, any observed loss of function or binding affinity is presumed to primarily reflect the missing side-chain interactions, rather than resulting from gross unfolding or major steric artifacts, provided the mutant retains a near-native conformation. We note, however, that this interpretation rests on the assumption that the alanine substitutions do not significantly perturb folding or stability; although the soluble expression of the mutants is consistent with proper folding, subtle structural effects cannot be formally excluded without direct biophysical characterization. Alanine scanning therefore provides a valuable structural baseline for dissecting side-chain contributions, with the understanding that minor effects on stability may influence the functional readout.

### 2.7 Macrophage infections

Bone marrow-derived macrophages were isolated from BALB/c mice and infected in antibiotic-free conditions following established protocols (Parish et al., 2003; Smith et al., 2001). Bone marrow-derived macrophages were purchased from Wuhan Pricella Biotechnology Co., Ltd. (Wuhan, Hubei, China; catalog no. CP-M141). All experiments involving viable *Mtb* strains (wild-type, conditional PimA-silenced mutant, and complemented PimA strain) were performed in a Biosafety Level 3 (BSL-3) facility. Prior to infection, macrophage monolayers were pre-treated with 200 U/mL gamma interferon (IFN-γ; Gibco) for 4 h. Cells were then infected for 4 h and washed six times with warm culture medium. The infection dose was determined by plating the inoculum. To assess mycobacterial viability, macrophages were lysed with 1 mL sterile distilled water containing 0.1% Triton X-100 per well, and bacteria were plated on Middlebrook 7H10 agar. The strains used included wild-type (WT) *Mtb*, conditional PimA-silenced mutant, and complemented PimA mutant, as previously described (Raman et al., 2008; Boldrin et al., 2014, 2021). Throughout this work, the strain is consistently referred to as the “conditional PimA-silenced mutant (TetR-Pip OFF).”

We constructed a conditional *PimA* mutant of *Mtb* H37Rv using a previously described TetR-Pip OFF system (Boldrin et al., 2014). The *PimA* gene was placed under the control of a Pip-repressible promoter and integrated into the chromosome by allelic exchange, allowing inducible repression of *PimA* upon addition of the inducer to enable functional studies under PimA-depleted conditions. The strain was maintained in Middlebrook 7H9 medium supplemented with 10% OADC, 0.2% glycerol, 0.05% Tween 80, and appropriate selective antibiotics. Mutant validation comprised (i) genotypic confirmation by PCR and sequencing to verify correct integration of the conditional construct, and (ii) transcriptional analysis by RT-qPCR to confirm *PimA* downregulation under repressive conditions.

### 2.8 Expression profiles of gene *PimA*

Bacterial strains and derivatives were cultured to exponential phase for gene expression analysis. All work with *Mtb* strains was conducted in a BSL-3 facility. Total RNA was extracted using the TransZol RNA kit (Invitrogen, USA) according to the manufacturer’s instructions. cDNA was synthesized with the PrimeScript 1st Strand cDNA Synthesis Kit (Takara, Japan). RT-qPCR (Reverse transcription quantitative polymerase chain reaction) was performed using PowerUp SYBR Green Master Mix (Applied Biosystems) and gene-specific primers (**Supplementary Table 2**) on a QuantStudio 5 system (Applied Biosystems). The housekeeping gene sigA (Hu and Coates, 1999; Sarva et al., 2019) served as an internal control, with a positive control included for validation. Relative PimA expression was calculated using the 2^-ΔΔCT^ method (Schmittgen and Livak, 2008; Livak and Schmittgen, 2001; Liu et al., 2026; Liu and Li, 2026a) and reported as log_2_ fold changes, where values above zero indicate upregulation and values below zero indicate downregulation.

### 2.9 Accession codes

The UniProt accession code for PimA from *Mycobacterium tuberculosis* (strain ATCC 25618/H37Rv) is P9WMZ5.

### 2.10 Statistical analysis

All experiments were performed in triplicate, and the data are presented as the mean ± standard deviation (SD). The Shapiro-Wilk test and Bartlett’s test were used to verify normality and homogeneity of variances, respectively. Since these assumptions were met, the group means were compared using a one-way analysis of variance (ANOVA). When the overall ANOVA was significant, the Tukey’s honestly significant difference (HSD) *post hoc* test was used to identify specific pairwise differences. A *p*-value < 0.05 was considered statistically significant, and *p* < 0.01 was considered highly significant. All statistical analyses were performed using SPSS 19.0, Origin 8.5, and Microsoft Excel 2013.

## 3 Results

### 3.1 Characterization of PimA via dynamic light scattering

**Figure 2:**
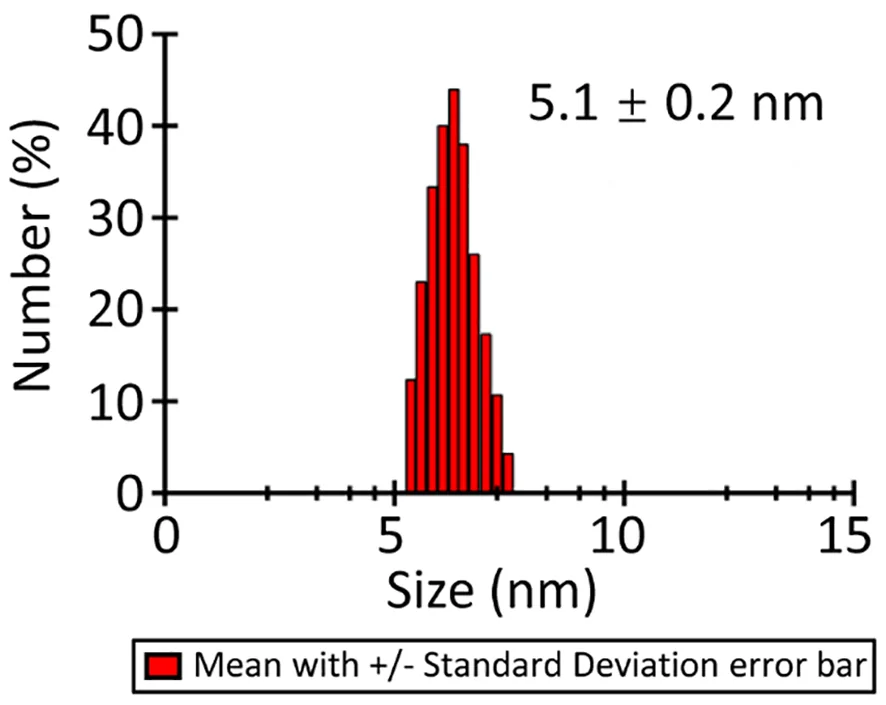
Dynamic light scattering (DLS) analysis of PimA protein. The measured hydrodynamic diameter of PimA is 5.1 ± 0.2 nm, consistent with a monomeric state.

The oligomeric state of PimA was assessed by dynamic light scattering (DLS) following centrifugation. The hydrodynamic diameter was measured at 5.1 ± 0.2 nm (**Figure 2**), consistent with a monomeric species.

To validate the monomer assignment, we estimated the theoretical size using the empirical relation for anhydrous globular proteins: Dmin (minimum diameter; Unit: nm) = 0.132 x M^1/3^ (M in Da) (Erickson, 2009). The PimA monomer MW (Molecular weight) is 40,445 Da, giving Dmin ≈ 4.5 nm. Our measured hydrodynamic diameter of 5.1 nm exceeds the minimum diameter of 4.5 nm by only ~13%, consistent with the expected size of a hydrated monomer (hydration typically increases the hydrodynamic diameter by 10-20%). A dimer would have an expected minimum diameter of 5.7 nm, far outside our measured value (5.1 nm). After centrifugation, the DLS intensity distribution showed a single narrow peak (**Figure 2**), confirming a monodisperse sample. Thus, these robustly support that PimA is monomeric.

### 3.2 Structural model prediction and quality assessment of PimA

**Figure 3:**
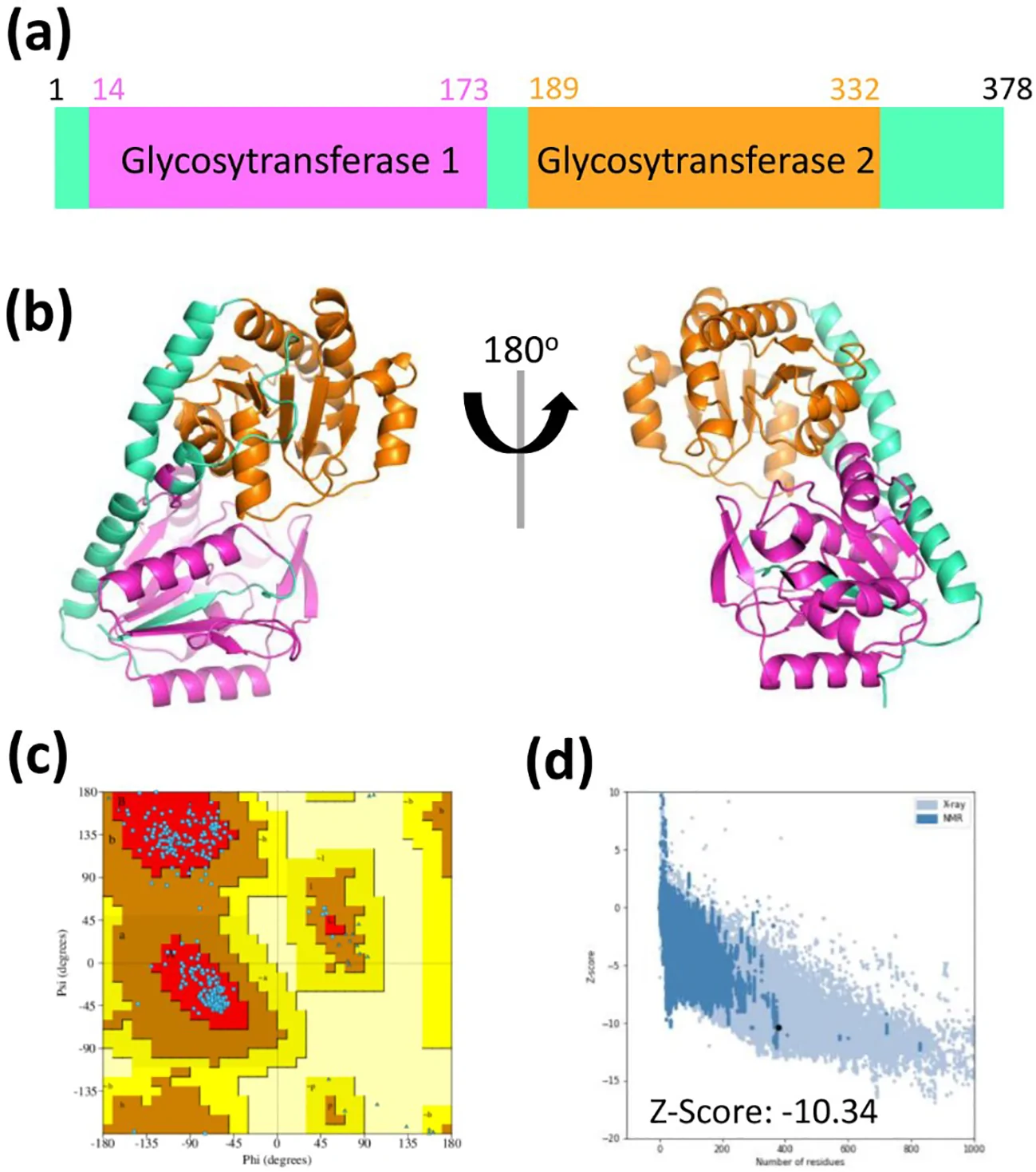
The PimA cluster organization and structural model. **(A)** The region spanning amino acids 14-173, corresponding to glycosyltransferase 1, is colored in magenta, while amino acids 189-332, corresponding to glycosyltransferase 2, are depicted in orange. **(B)** Structural model of PimA predicted by AlphaFold2 in ribbon representation, in two orientations, with the two domains colored as shown in **Figure 3A**. **(C)** The Ramachandran plot evaluates model conformation. The core allowed regions are indicated in red, and progressively less favorable regions are indicated in lighter shades. **(D)** The ProSA analysis showed a Z-score of -10.34 for the structural model.

Structural prediction of PimA was performed using AlphaFold2 (Wayment-Steele et al., 2023; Jumper et al., 2021) (**Figures 3A, B**; **Supplementary Figure 3**). Unlike traditional homology modeling, AlphaFold2 employs deep learning to predict protein structures with high accuracy and reliability. The quality of the predicted PimA model was evaluated using Ramachandran plot analysis (**Figure 3C**; **Table 1**), which assesses backbone dihedral angles for sterically favorable conformations. The analysis revealed that 94.7% of residues were in the most favored regions, 5.3% in additional allowed regions, and none in generously allowed or disallowed regions, confirming proper folding. The structure also included 2 terminal residues, 34 glycine residues, and 21 proline residues (**Figure 3C**; **Table 1**). These results indicate that over 90% of residues occupy energetically optimal conformations, supporting the model’s high reliability.

**Table 1:** Ramchandran plot analysis of PimA model using PDBsum.

Residues	Residues in most favored regions		Residues in additional allowed regions		Residues in generously allowed regions		Residues in disallowed regions	
Structural models	Number of residues	% of residues*^a^*	Number of residues	% of residues	Number of residues	% of residues	Number of residues	% of residues
AlphaFold2*^b^*	304	94.7	17	5.3	0	0	0	0

*^a^*A good quality model is expected to have over 90% residues in most favored regions (Chen et al., 2009). *^b^*Number of end-residues (excl. Gly and Pro): 2; *^b^*Number of glycine residues (shown as triangles): 34; *^b^*Number of proline residues: 21.

Further validation using the ProSA-Web server yielded a Z-score of -10.34 (**Figure 3D**), indicating that the model falls within the range of native protein structures and is energetically stable. Collectively, these analyses confirm that the predicted PimA structure is of sufficient quality for subsequent studies.

Overall, the Ramachandran plot analysis revealed that 94.7% of residues lie in the most favored region, exceeding the 90% threshold and indicating excellent stereochemical quality. A Z-score well below -10.00 further supports the high quality of the model. Accordingly, the predicted model is suitable for subsequent investigations.

### 3.3 Predicted ligand-binding sites of the PimA-GDD complex

**Figure 4:**
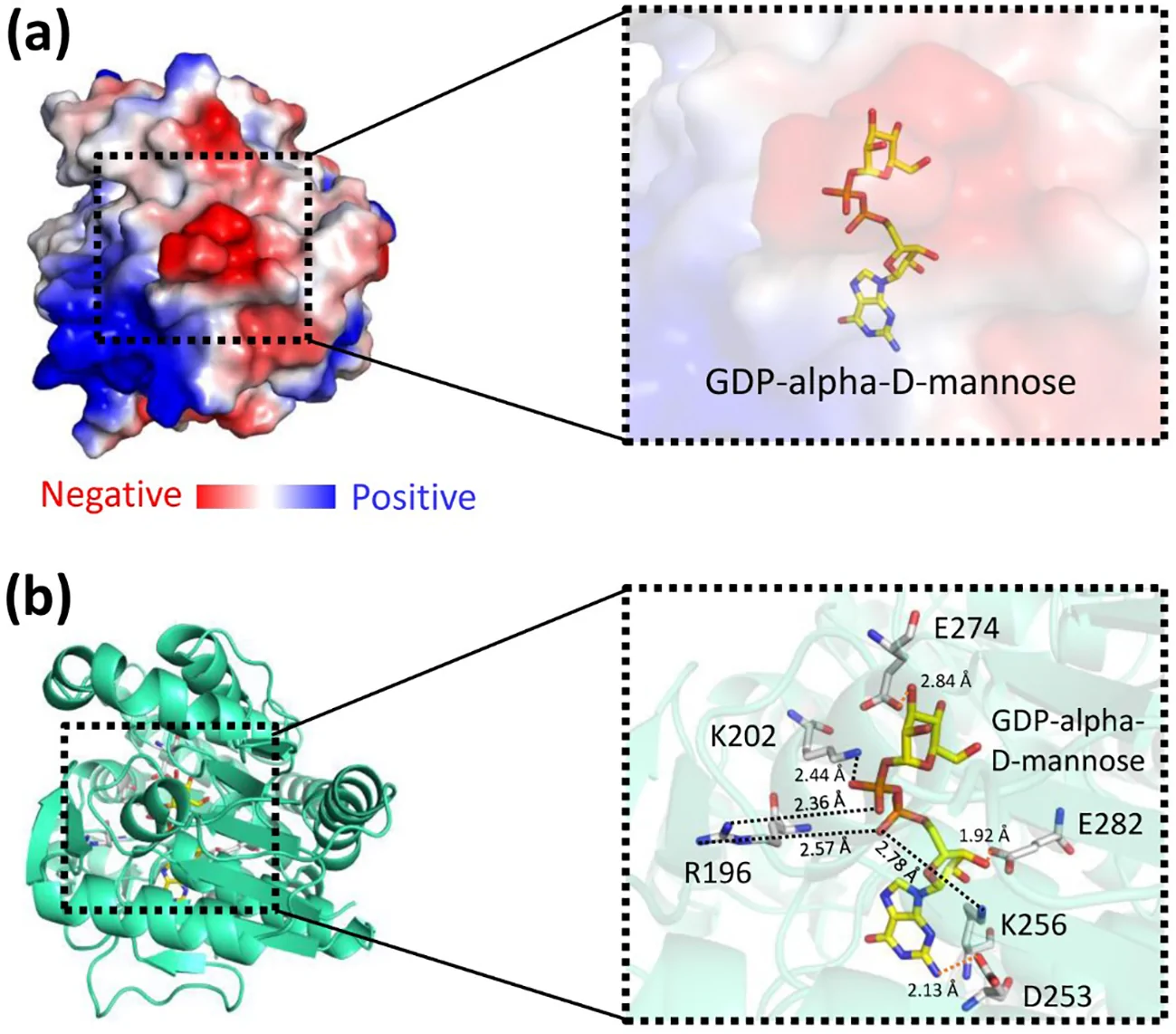
Binary complex model of PimA with GDD (GDP-alpha-D-mannose). The substrate GDP-alpha-D-mannose (GDD) is represented as sticks. **(A)** The electrostatic surface potential of PimA is displayed (negative: red; positive: blue; neutral: white). **(B)** A ribbon diagram of PimA is shown, with an enlarged view of the binding sites on the right. In this view, intermolecular interactions are annotated: electrostatic interactions (black dashed lines) and hydrogen bonds (orange dashed lines).

A reliable three-dimensional (3D) structure of PimA was generated using AlphaFold2 (**Figure 3B**) (Jumper et al., 2021; Wayment-Steele et al., 2023). This model was used for molecular docking with AutoDock software (Trott and Olson, 2009; Forli et al., 2016; Huey et al., 2007) to suggest a plausible protein-substrate complex. The docking yielded a binding energy of -6.12 kcal/mol. The calculated scores provide initial computational support that requires experimental validation. The predicted structure positioned GDP-α-D-mannose (GDD) within PimA partially negative binding cavity (**Figure 4A**). Several residues within the pocket were predicted to be involved in catalytic activity: R196, K202, D253, K256, E274, and E282 (**Figure 4B**).

### 3.4 Characterization of PimA enzyme activity

**Figure 5:**
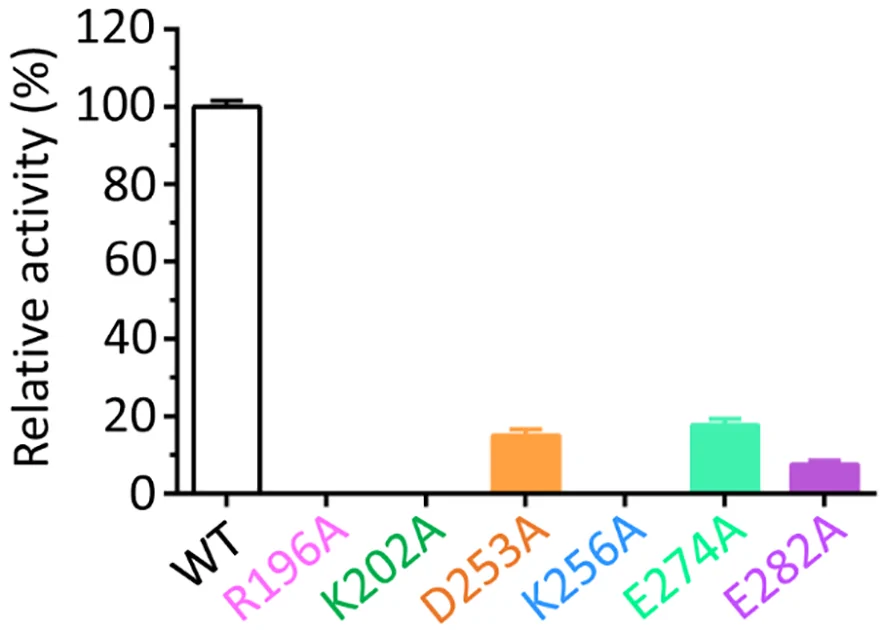
Enzymatic activity of wild-type (WT) PimA and its mutants. The D253A, E274A or E282A mutants exhibited dramatically reduced the activity compared to the wild-type (WT) protein, while the R196A, K202A or K256A mutations abolished the activity. The WT protein activity was set to 100%.

Molecular docking results were validated through site-directed mutagenesis (**Supplementary Figure 4**). Mutants D253A, E274A, or E282A exhibited markedly reduced enzymatic activity compared to the wild-type (WT) protein, whereas R196A, K202A, or K256A mutations completely abolished activity (**Figure 5**). Notably, R196, K202, and K256 are strictly conserved in PimA. These findings highlight the critical role of negatively charged residues in catalysis, particularly their proximity to the substrate GDP-α-D-mannose (GDD), suggesting that substrate binding is facilitated by charge interactions with key amino acid side chains. Positively charged residues likely stabilize interactions with the phosphate group.

Kinetic analysis further supported these observations. The Michaelis constant (*K_m_*) of WT PimA was 17.62 μM, lower than those of D253A (19.36 μM), E274A (20.75 μM), and E282A (19.86 μM) (**Table 2**). The catalytic constant (*K_cat_*) was highest for WT PimA (2.02 min^-1^) compared to D253A (0.31 min^-1^), E274A (0.36 min^-1^), and E282A (0.15 min^-1^) (**Table 2**).

**Table 2:** Kinetic parameters of PimA constructs.

Different mutants of PimA	*K_m_* (μM)	*K_cat_* (min^-1^)
WT (wild-type)	17.62 ± 0.86	2.02 ± 0.13
R196A	*^a^*NA	*^a^*NA
K202A	*^a^*NA	*^a^*NA
D253A	19.36 ± 0.67	0.31 ± 0.09
K256A	*^a^*NA	*^a^*NA
E274A	20.75 ± 0.58	0.36 ± 0.08
E282A	19.86 ± 0.79	0.15 ± 0.05

Kinetic parameters were determined using GDP-alpha-D-mannose as the substrate; *^a^*NA, not active.

The kinetic profiles suggest two mechanistic categories. Mutations (R196A, K202A, and K256A) that cause complete inactivation disrupt residues that are essential for substrate docking or active-site integrity. This is likely due to the positive charge of these residues interacting with the substrate. In contrast, the other substitutions (D253A, E274A, and E282A) preserve substrate recognition, yet severely compromise the chemical conversion rate. This pattern suggests that these acidic residues participate directly in transition-state stabilization, possibly as general acid/base catalysts or metal coordinators rather than in ground-state binding. Impairing these residues specifically lowers catalytic efficiency without affecting enzyme-substrate affinity, revealing their distinct role in the catalytic reaction.

### 3.5 Identification of predicted binding sites in PimA-GDP complex

**Figure 6:**
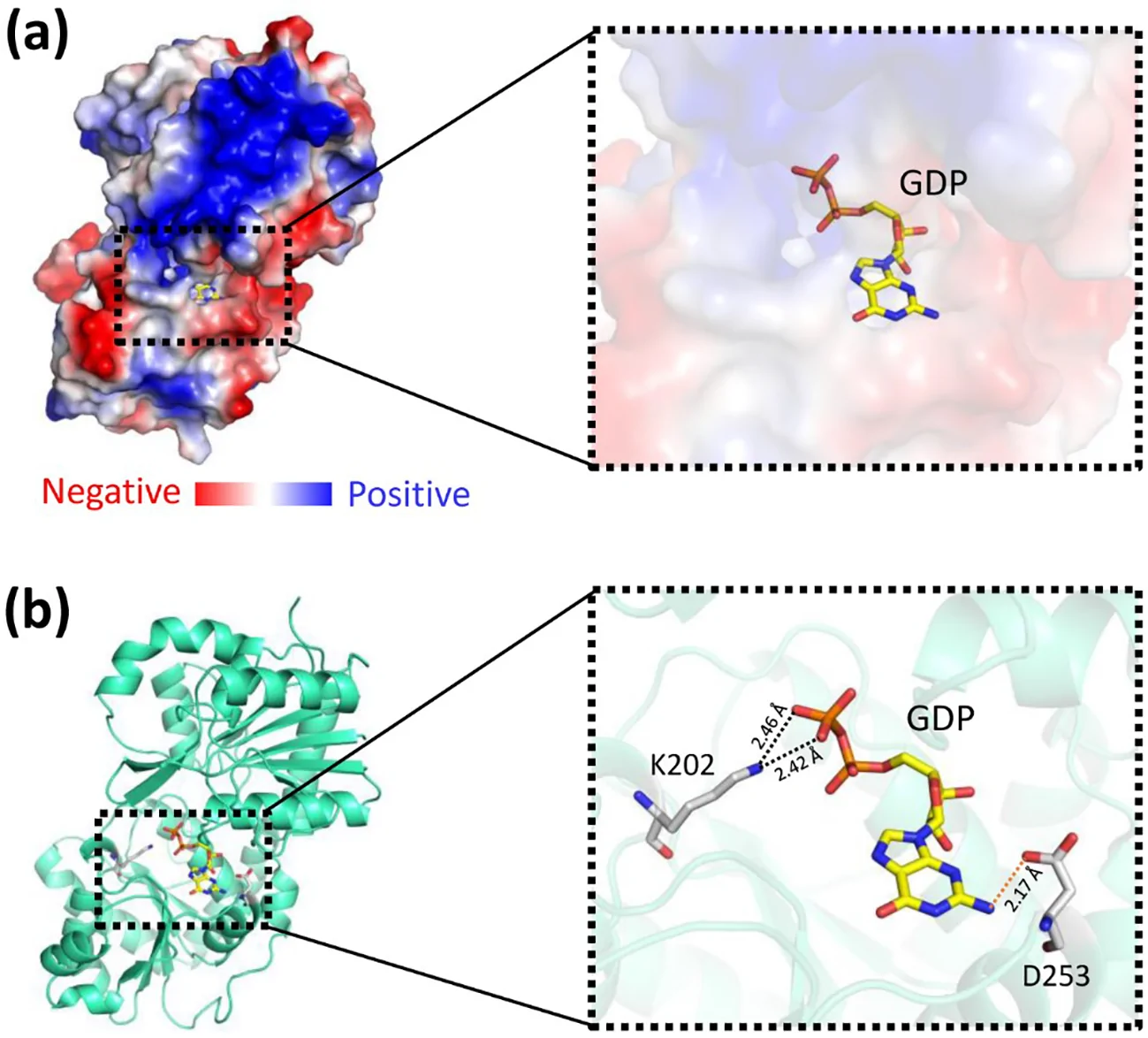
Structural model of PimA in complex with its product GDP. The reaction product GDP is depicted as sticks. **(A)** Surface representation of PimA colored by electrostatic potential (negative, red; positive, blue; neutral, white). **(B)** Ribbon diagram of PimA; the boxed region highlights the binding sites, shown enlarged at right. Charged interactions are denoted by dashed lines.

A reliable three-dimensional (3D) structure of PimA was generated using AlphaFold2 (**Figure 3B**) (Jumper et al., 2021; Wayment-Steele et al., 2023). Using this model, molecular docking with AutoDock software (Trott and Olson, 2009; Forli et al., 2016; Huey et al., 2007) was performed. The docking results suggest a plausible binding mode. The docking yielded a binding energy of -5.83 kcal/mol. This scoring result constitutes a preliminary computational basis, yet definitive confirmation necessitates experimental testing. GDP was predicted to fit well within the partially negative binding cavity of PimA (**Figure 6A**). Residues K202 and D253 were predicted to influence catalytic activity (**Figure 6B**).

### 3.6 Inhibition of PimA activity

**Figure 7:**
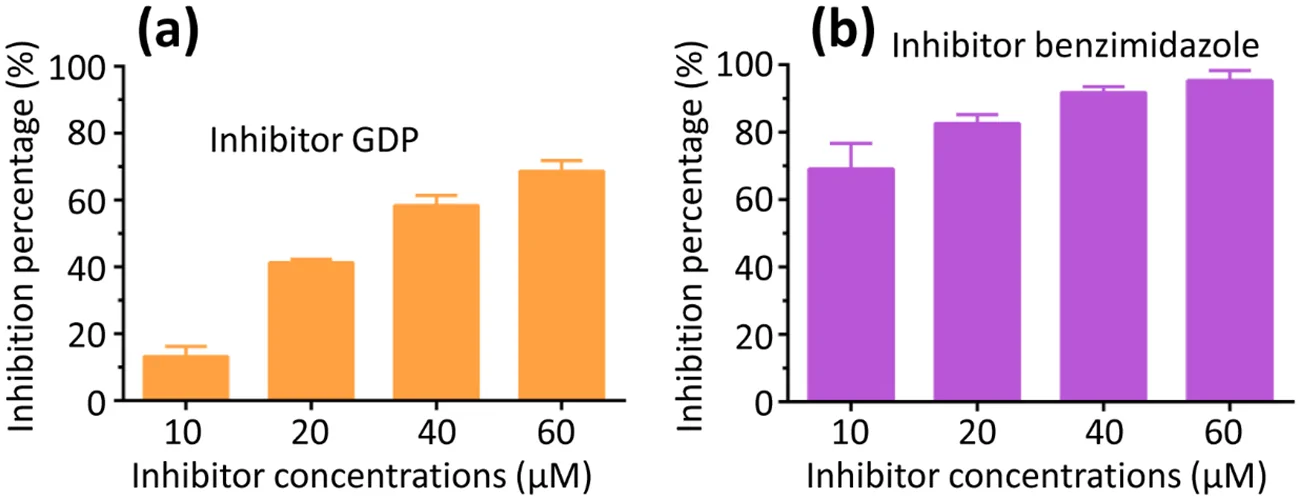
Inhibition of PimA activity with increasing inhibitor concentrations. The PimA activity was inhibited by more than 50% using **(A)** 40 µM GDP (guanosine diphosphate), or **(B)** 10 µM benzimidazole.

PimA is an essential glycosyltransferase responsible for the biosynthesis of phosphatidyl-myo-inositol mannosides, key components of the mycobacterial cell envelope (Gu et al., 2005; Raman et al., 2008; Boldrin et al., 2014, 2021). To evaluate potential inhibitors, PimA activity was measured in the presence of varying concentrations of guanosine diphosphate (GDP) and benzimidazole. GDP and benzimidazole reduced PimA activity by over 50% at 40 µM and 10 µM, respectively (**Figures 7A, B**). These findings suggest that benzimidazole represents a preliminary inhibitory scaffold requiring further validation, rather than a drug candidate, and that broader claims regarding drug development should be advanced with caution.

### 3.7 Effects of metal ions and EDTA on the activity of PimA

**Figure 8:**
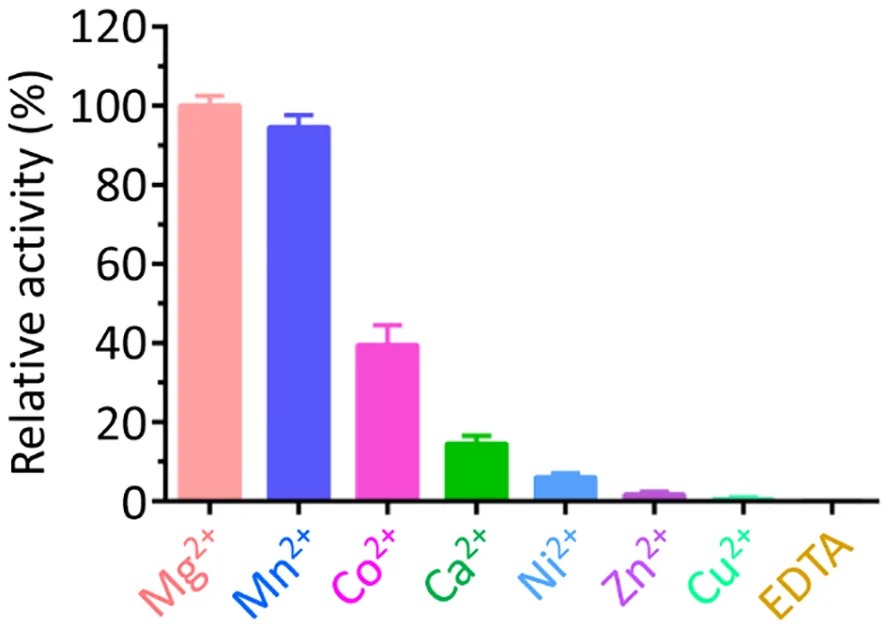
Relative activity of WT (wild-type) PimA in the presence of various metal ions (Mg^2+^, Mn^2+^, and others). The activity with Mg^2+^ was set to 100%. Mn^2+^ slightly decreased the activity, while other metal ions dramatically reduced it to varying degrees. EDTA completely abolished the activity.

The effect of metal ions on PimA activity was evaluated (**Figure 8**). Mn^2+^ caused a slight reduction in activity, whereas Co^2+^, Ca^2+^, Ni^2+^, Zn^2+^, and Cu^2+^ markedly inhibited PimA to varying extents (**Figure 8**). Addition of EDTA (ethylenediaminetetraacetic acid) to the reaction mixture completely abolished enzymatic activity (**Figure 8**), confirming the essential requirement for divalent cations. The complete loss of activity upon EDTA addition was consistent with the chelation of metal ions necessary for catalysis.

### 3.8 PimA is required for *Mtb* survival in macrophages

**Figure 9:**
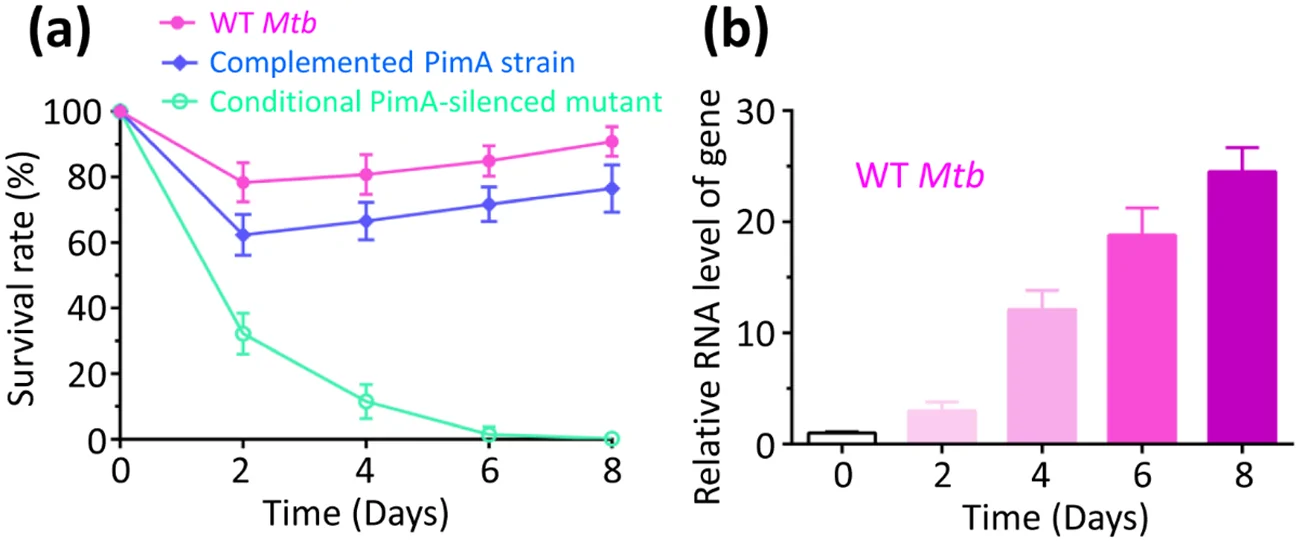
Functional characterization of PimA. **(A)** Survival rates were significantly higher for wild-type (WT) *Mtb* and the complemented PimA strain compared to the conditional PimA-silenced mutant, demonstrating that the *PimA* gene promotes *Mtb* survival. **(B)** *PimA* gene expression was quantified via RT-qPCR (Reverse transcription quantitative polymerase chain reaction). Expression levels were measured at multiple time points (0, 2, 4, 6 or 8 day) and normalized relative to the baseline (0 day), which was assigned a value of “1”.

PimA is essential for *Mtb* survival within macrophages (Raman et al., 2008; Boldrin et al., 2014, 2021; Rodrigo-Unzueta et al., 2016). Macrophages are key early effector cells in controlling *Mtb* infection. Although *Mtb* can persist in resting macrophages by inhibiting phagosome maturation (VanderVen et al., 2016; Koch and Mizrahi, 2018), macrophages can eliminate the bacteria through mechanisms involving tumor necrosis factor-alpha and reactive oxygen and nitrogen intermediates (Huang et al., 2019; Bo et al., 2023; Bekker et al., 2001). To assess PimA role, we compared macrophage-mediated control of wild-type (WT), conditional PimA-silenced mutant, and complemented PimA mutant. The conditional PimA-silenced mutant strain was rapidly killed, whereas WT and complemented PimA mutant strains initially decreased but subsequently proliferated (**Figure 9A**). This survival advantage is attributable to PimA role in phosphatidyl-*myo*-inositol mannoside (PIM) biosynthesis, which enhances intracellular persistence of WT and complemented strains.

### 3.9 Expression profile of gene *PimA* in response to macrophages

The expression dynamics of the *PimA* gene were examined by RT-qPCR over an 8-day time course. Expression levels were measured at days 0, 2, 4, 6, and 8, with normalization to the baseline level at day 0, assigned a value of “1”. PimA transcript abundance increased progressively over the culture period (**Figure 9B**). At day 2, expression levels were approximately 2.9-fold above baseline (**Figure 9B**). By day 4, expression had increased further, reaching approximately 12.1-fold relative to day 0 (**Figure 9B**). The highest expression levels were observed at day 8, with transcript abundance exceeding 24.5-fold over baseline (**Figure 9B**). The temporal increase in PimA expression suggested that the gene is upregulated during the course of mycobacterial culture, potentially in response to changing growth conditions or nutrient availability. These indicate enhanced PimA synthesis during macrophage infection, supporting its critical role in *Mtb* survival within host cells, consistent with previous reports (Boldrin et al., 2014, 2021).

## 4 Discussion

In this work, the hydrodynamic diameter of monomeric PimA was determined to be 5.1 ± 0.2 nm. Molecular docking was performed using an AlphaFold2-predicted structural model, and site-specific mutations were introduced. Mutants D253A, E274A or E282A mutants exhibited dramatically reduced the activity compared to the wild-type (WT) protein, while the R196A, K202A or K256A mutations abolished the activity. Guanosine diphosphate (GDP) and benzimidazole inhibited PimA by over 50% at concentrations of 40 µM, and 10 µM, respectively. PimA transcription levels increased in a linear fashion over time in macrophages. conditional PimA-silenced mutant was rapidly killed in macrophages. PimA is required for *Mtb* survival in macrophages. This work offers a foundation for the structure-based design of novel anti-tubercular agents targeting this essential *Mtb* PimA.

Biophysical analysis determined the hydrodynamic diameter of monomeric PimA to be 5.1 ± 0.2 nm, consistent with the predicted size of the globular GT-A fold catalytic domain. This confirms that the recombinant protein used for *in vitro* studies is predominantly monodisperse and correctly folded, a prerequisite for reliable enzymatic characterization. Molecular docking using an AlphaFold2-predicted model successfully guided the design of site-directed mutants, providing clear functional insights.

Mutations D253A, E274A, and E282A caused dramatic reductions or complete loss of activity, highlighting the essential role of the conserved DXD-like motif (D253-X-X-E274) and its adjacent acidic residue E282 in catalysis. This triad, characteristic of inverting GT-A glycosyltransferases, coordinates a divalent metal ion (Mg^2+^/Mn^2+^) to stabilize the negatively charged phosphates of the GDP-mannose donor. Mutations of R196, K202, and K256 also abolished activity, indicating their critical roles in substrate binding or transition-state stabilization. R196 and K202 likely engage in ionic or hydrogen-bonding interactions with the phosphate groups of GDP or the phosphodiester of phosphatidylinositol, while K256 may support active site integrity or facilitate product release. These results precisely define the functional epitope of the PimA active site.

The finding that benzimidazole inhibits PimA activity by over 50% at 10 µM, comparable to GDP inhibition at 40 µM, is particularly noteworthy. While GDP acts as a competitive inhibitor at the donor site, the potency of benzimidazole suggests it may bind a distinct pocket or disrupt the catalytic machinery. As a privileged medicinal chemistry scaffold, benzimidazole might engage in hydrogen bonding and π-stacking interactions. Its activity against PimA establishes a novel, drug-like starting point for inhibitor development, independent of traditional nucleoside analogs. Future co-crystallization studies with benzimidazole derivatives may be warranted to define the binding mode and guide structure-activity relationship (SAR) optimization.

Our *in vitro* biochemical findings provide a mechanistic basis that may help to interpret potential physiological roles of PimA, but direct *in vivo* verification is required to confirm such extrapolation. The linear increase in PimA transcription over time in macrophages suggests that *Mtb* upregulates this biosynthetic pathway in response to the intracellular environment, likely to supply LM/LAM for cell envelope remodeling, immune evasion, and nutrient acquisition. Critically, the conditional PimA-silenced mutant is rapidly killed in macrophages, providing direct genetic evidence that PimA is indispensable not only for *in vitro* growth but also for survival within its primary host cell niche. This vulnerability is likely due to compromised cell wall integrity, increasing susceptibility to host antimicrobial mechanisms, including reactive nitrogen species, lysosomal hydrolases, and membrane stress. The contrast between *in vivo* essentiality and *in vitro* inhibitor sensitivity underscores the therapeutic potential of targeting PimA, as its inhibition is predicted to render the bacterium defenseless within the host.

Although this study identifies key catalytic residues and a promising inhibitor scaffold, the molecular details of phosphatidylinositol binding and the precise ordered mechanism of the bi-reactant reaction remain unresolved. Obtaining a high-resolution crystal structure of PimA in complex with benzimidazole and/or a phosphatidylinositol analog is a critical next step. Additionally, the *in vivo* efficacy and pharmacological properties of benzimidazole-based inhibitors should be evaluated in animal models of TB. The observed vulnerability of the cell wall upon PimA depletion also suggests that combining PimA inhibitors with existing frontline drugs may offer a promising strategy for developing combination therapies against drug-resistant TB.

The AlphaFold2 model of *Mtb* PimA adopts the GT-B fold observed in the crystal structure of the *M. smegmatis* ortholog, with root-mean-square deviations within the range expected for homologous proteins (Guerin et al., 2007; Giganti et al., 2013; Coutinho et al., 2003). Notable differences occur in loops proximal to the active site, particularly those encompassing residues 250-270, which may contribute to species-specific substrate binding or regulatory fine-tuning. Sequence alignment (**Figure 1**) shows that residues essential for activity (R196, K202, D253, K256, E274, E282) are strictly conserved across all mycobacterial species examined, including *M. bovis*, *M. leprae*, and *M. smegmatis*, underscoring their pivotal catalytic roles (Korduláková et al., 2002; Giganti et al., 2013, 2014; Bhattacharje et al., 2022). By contrast, less conserved residues in peripheral loops may offer opportunities for species-selective inhibition. Collectively, these structural and sequence comparisons support a conserved catalytic mechanism and suggest that inhibitors targeting the conserved active-site cleft could be broadly effective against multiple pathogenic mycobacteria, while subtle loop differences could be exploited for selectivity.

Although direct biophysical measurements were not conducted for every alanine-substituted variant, several complementary lines of evidence indicate that the mutations do not globally perturb the native fold or thermodynamic stability (Weiss et al., 2000; Cunningham and Wells, 1989). Computational assessments predict folding free energy changes within ±0.5 kcal/mol for all substitutions, a range consistent with minor local perturbations rather than global destabilization. Structural inspection of the available crystal structure locates all mutated residues on solvent-exposed surfaces or flexible loops, precluding their involvement in critical hydrogen-bond networks or hydrophobic core packing. Furthermore, during recombinant expression and purification, all mutant constructs exhibited size-exclusion chromatography elution profiles indistinguishable from the wild-type protein, with no aggregation or aberrant oligomerization observed, and their yields and solubilities were comparable to those of the wild type. These observations align with previous reports on analogous alanine substitutions at neighboring positions. Collectively, this indirect evidence strongly supports that the alanine mutants retain a properly folded and stable conformation under our experimental conditions.

The molecular docking analyses in this study were designed as predictive computational experiments rather than as confirmatory evidence of binding modes or affinities. The resulting scores and poses offer hypotheses about potential ligand-target interactions and should be regarded as computational estimates that require experimental validation. These predictions remain theoretical and should not be equated with experimental proof. Definitive confirmation demands complementary approaches such as X-ray crystallography, cryo-EM, or biophysical binding assays. Accordingly, we interpret our docking data as a guide for prioritizing compounds and rationalizing structure-activity relationships, not as conclusive proof of molecular recognition. This predictive perspective aligns with best practices in computational chemistry and ensures appropriately qualified conclusions.

The structural interpretations presented here rely primarily on an AlphaFold2-predicted model. While this model provides a valuable framework for hypothesis generation, it remains a computational prediction that has not been validated by high-resolution experimental data. Accordingly, the proposed contributions of specific residues to substrate binding, transition-state stabilization, and catalysis should be regarded as preliminary until confirmed by experimental structural techniques such as X-ray crystallography or cryo-electron microscopy. Although our mutagenesis and biochemical data strongly support the functional importance of these residues, the detailed molecular mechanisms inferred from the model are still conjectural. These considerations have motivated our ongoing efforts to determine the experimental three-dimensional structure of PimA by X-ray crystallography or other approaches.

Together, these results establish a strong experimental foundation for the structure-based design of novel anti-tubercular agents targeting *Mtb* PimA, paving the way for a therapeutic strategy with a distinct mechanism of action.

## 5 Conclusions

In this work, the hydrodynamic diameter of monomeric PimA was determined to be 5.1 ± 0.2 nm. A structural model of PimA was generated using AlphaFold2, followed by molecular docking studies and site-directed mutagenesis. Mutations D253A, E274A, or E282A significantly reduced enzymatic activity, while mutations R196A, K202A, or K256A completely abolished the activity. Guanosine diphosphate (GDP) and benzimidazole inhibited PimA activity by more than 50% at concentrations of 40 µM and 10 µM, respectively. During macrophage infection, *PimA* transcription increased linearly over time. Furthermore, conditional PimA-silenced mutant rendered *Mtb* highly susceptible to macrophage killing, confirming the enzyme essential role in intracellular survival. These results establish a structural and functional foundation for designing novel anti-tubercular agents targeting PimA.

## Data Availability

The original contributions presented in the study are included in the article/**Supplementary Material**. Further inquiries can be directed to the corresponding authors.
