# Supplementary material for: Functional and mechanistic characterization of the phosphatidyl-myo-inositol mannosyltransferase PimA from Mycobacterium tuberculosis: implications for anti-tuberculosis drug development
Source: Front Cell Infect Microbiol. 2026 Aug 19;16:1888373. doi: 10.3389/fcimb.2026.1888373 (PMC13533801; doi:10.3389/fcimb.2026.1888373)
Supplement: Supplementary file 1 [file DataSheet1.pdf]

Supplementary Material

Functional and mechanistic characterization of the phosphatidyl-*myo*-inositol mannosyltransferase PimA from *Mycobacterium tuberculosis*: implications for anti-tuberculosis drug development

Dafeng Liu[1,2,*], Huashui Deng[2], Xinbao Liu[2], Wenshuang Yao[2] and Na Li[1,*]

[1]College of Biological Sciences and Technology, Yili Normal University, Yining 835000, Xinjiang, China;

[2]School of Life Sciences, Xiamen University, Xiamen 361102, Fujian, China.

[*]Correspondence: dafeli@sina.cn or liudafeng2017@163.com (Dafeng Liu); nali2026@qq.com (Na Li).

Figure S1.

MRIGMICPYSFDVPGGVQSHVLQLAEVMRTRGHLVSVLAPASPHAALPDYFVSGGRAVPIPYNGSVARLRFGP
ATHRKVKKWLAHGDFDVLHLHEPNAPSLSMLALNIAEGPIVATFHTSTTKSLTLTVFQGILRPMHEKIVGRIAVSD
LARRWQMEALGSDAVEIPNGVDVDSFASAARLDGYPRQGKTVLFLGRYDEPRKGMAVLLDALPKVVQRFPDV
QLLIVGHGDADQLRGQAGRLAAHLRFLGQVDDAGKASAMRSADVYCAPNTGGESFGIVLVEAMAAGTAVVA
SDLDAFRRVLRDGEVGHLVPVDPPDLQAAALADGLIAVLENDVLRERYVAAGNAAVRRYDWSVVASQIMRVY
ETVAGSGAKVQVAS

Figure S1. The amino acid sequence of PimA (UniProt code P9WMZ5).

Figure S2.

ATGCGTATCGGCATGATTTGTCCATACTCTTTTGATGTTCCGGGTGGCGTCCAGTCTCATGTGCTGCAGCTGG
CGGAAGTTATGCGCACTCGTGGTCACCTGGTATCCGTGCTGGCACCGGCGAGCCCGCACGCTGCACTGCCG
GACTACTTCGTTAGCGGTGGCCGTGCGGTACCGATTCCGTATAATGGCTCTGTGGCTCGCCTGCGCTTCGGC
CCGGCAACTCACCGCAAGGTTAAGAAATGGCTGGCTCATGGTGACTTCGACGTTCTGCATCTGCACGAACC
GAACGCGCCGAGCCTGAGCATGCTGGCTCTGAACATCGCTGAAGGTCCGATCGTCGCCACTTTCCACACGA
GCACGACCAAATCTCTGACCCTGACCGTATTCCAGGGTATTCTGCGCCCGATGCACGAAAAAATCGTGGGCC
GTATCGCTGTGAGCGATCTGGCCCGTCGTTGGCAAATGGAAGCACTGGGCAGCGACGCTGTTGAAATCCCG
AACGGTGTGGATGTTGACTCCTTCGCAAGCGCGGCGCGCCTGGATGGTTACCCGCGTCAGGGTAAAACCGT
GCTGTTCCTGGGTCGTTATGACGAACCGCGTAAGGGTATGGCTGTGCTGCTGGATGCTCTGCCGAAAGTTGT
GCAGCGTTTCCCGGACGTACAGCTGCTGATTGTTGGTCACGGTGACGCTGACCAGCTGCGTGGTCAGGCAG
GTCGTCTGGCTGCTCACCTGCGCTTTCTGGGTCAGGTTGACGATGCAGGCAAGGCATCCGCAATGCGTTCT
GCCGACGTTTATTGCGCGCCGAACACCGGCGGCGAGTCCTTCGGTATCGTACTGGTTGAAGCCATGGCTGC
AGGTACTGCTGTGGTTGCTTCTGACCTGGACGCGTTCCGCCGTGTTCTGCGTGATGGTGAAGTTGGTCATCT
GGTACCAGTGGACCCGCCAGACCTGCAGGCAGCGGCACTGGCCGACGGTCTGATCGCCGTGCTGGAAAAC
GATGTGCTGCGTGAACGTTACGTTGCTGCGGGTAACGCTGCGGTGCGCCGTTACGATTGGTCTGTTGTCGC
GTCTCAGATTATGCGTGTCTATGAAACGGTTGCCGGCAGCGGTGCAAAAGTTCAGGTGGCCTCC

Figure S2. The optimized gene sequence of *PimA*.

Figure S3.

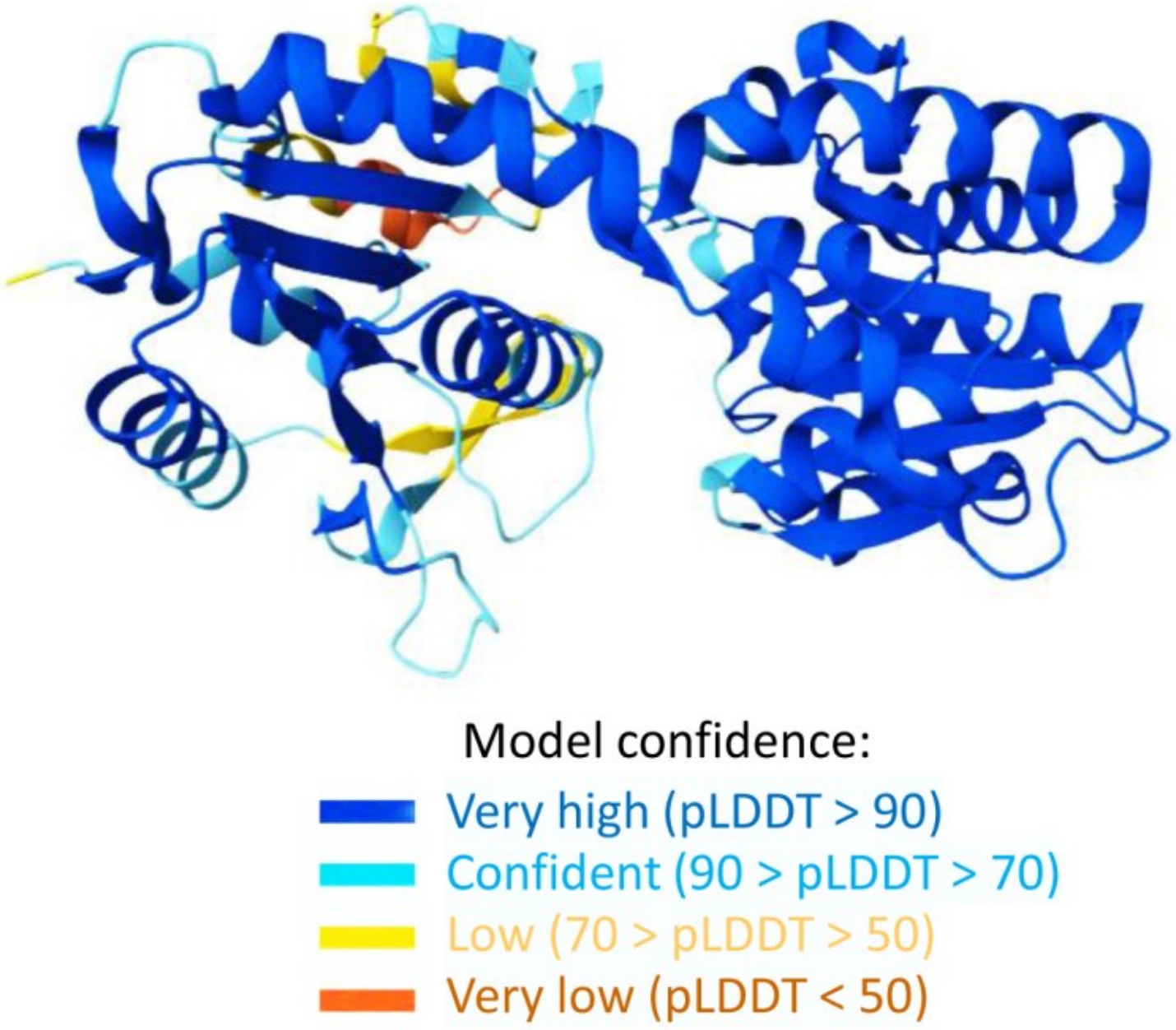

Figure S3. The confidence of the AlphaFold2-predicted PimA structure was assessed via the per-residue Local Distance Difference Test (pLDDT). This metric ranges from 0 to 100, with higher values denoting greater predicted local accuracy.

Figure S4.

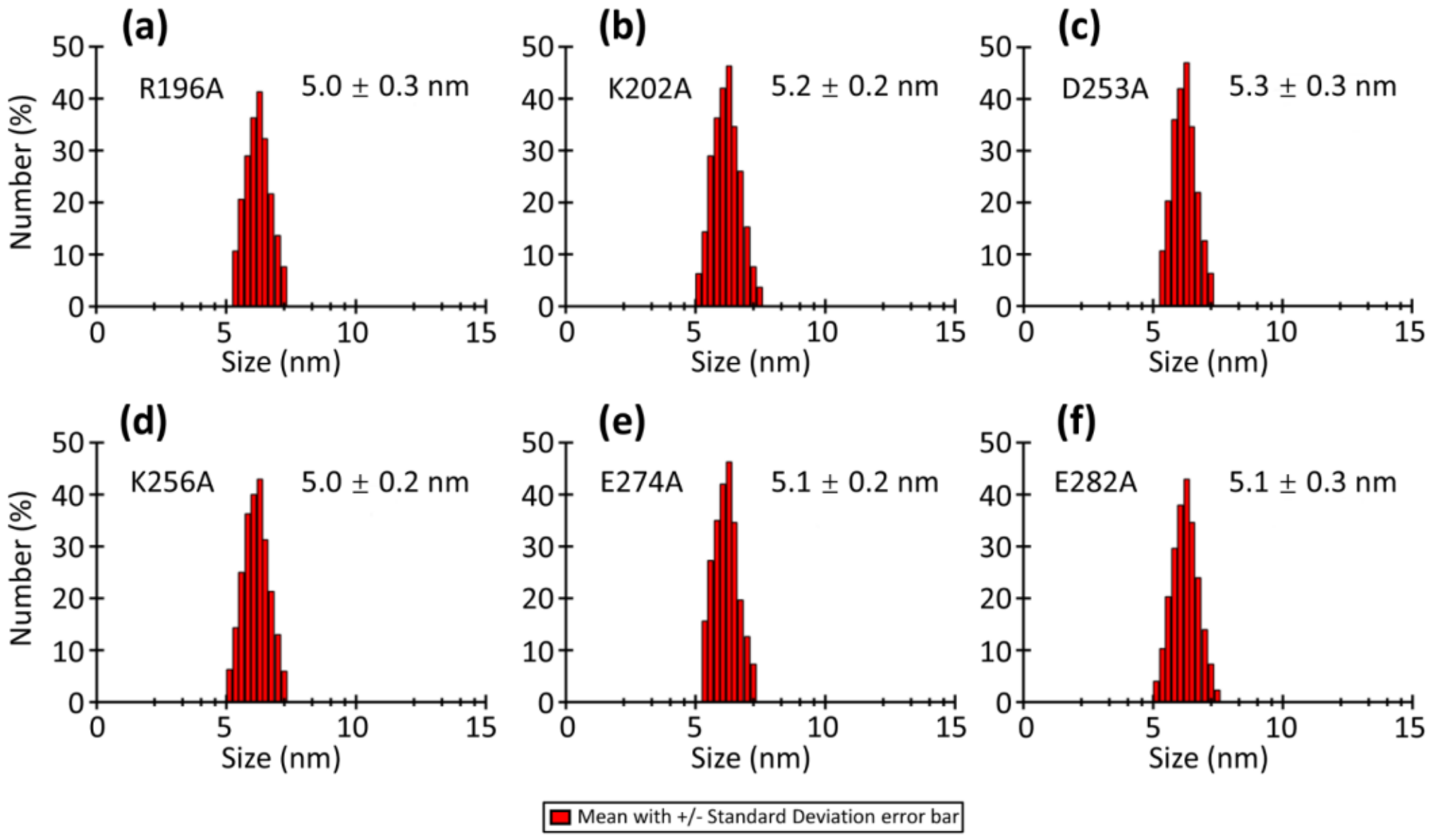

Figure S4. Dynamic light scattering (DLS) analysis of different PimA mutants. The hydrodynamic diameters of mutants R196A, K202A, D253A, K256A, E274A, and E282A were (a) 5.0 ± 0.3 nm, (b) 5.2 ± 0.2 nm, (c) 5.3 ± 0.3 nm, (d) 5.0 ± 0.2 nm, (e) 5.1 ± 0.2 nm, and (f) 5.1 ± 0.3 nm, respectively, showing that all mutants remain in a monomeric state.

Table S1.

Table S1. Primers used for generating site-directed mutants of PimA

Primers	Primer sequence (5'-3')
R196A (F)	GTTCCTGGGT**gca**TATGACGAACCGCG
R196A (R)	CGTCATA**tgc**ACCCAGGAACAGCACGG
K202A (F)	CCGCGT**gca**GGTATGGCTGTGCTGCTG
K202A (R)	GCCATACC**tgc**ACGCGGTTCGTCATAACGACC
D253A (F)	GGTTGAC**gca**GCAGGCAAGGCATCCGC
D253A (R)	CTTGCCTGC**tgc**GTCAACCTGACCCAGAAAGC
K256A (F)	GCAGGC**gca**GCATCCGCAATGCG
K256A (R)	GCGGATGC**tgc**GCCTGCATCGTCAACC
E274A (F)	GGCGGC**gca**TCCTTCGGTATCGTACTGG
E274A (R)	CCGAAGGA**tgc**GCCGCCGGTG
E282A (F)	GTACTGGTT**gca**GCCATGGCTGCAGGTACTGCTG
E282A (R)	GCCATGGC**tgc**AACCAGTACGATACCGAAGG

Note: Mutagenic regions of the sequence are shown in underlined and bold.

Table S2.

Table S2. Primers used for RT-qPCR

Primers	Primer sequence (5'-3')
SigA (F)	gtggcagcgaccaaagcaag
SigA (R)	tcagtccaggtagtcgtgc
PimA (F)	atgcggatcggcatgatttgtccgtactcg
PimA (R)	tcagctggccacctgaaccttggc
